# Supplementary material for: Development of a computer-aided design software for the quantitative evaluation of aesthetic damage
Source: PLoS One. 2019 Dec 18;14(12):e0226322. doi: 10.1371/journal.pone.0226322 (PMC6919621; doi:10.1371/journal.pone.0226322)
Supplement: S1 Code site — 1. (ZIP) [file pone.0226322.s001.zip › [EESC_jr][Projeto]Relatorio_07_21/reports/templates_zip/gentelella-master/vendors/morris.js/examples/stacked_bars.html]

# Stacked Bars chart

```
// Use Morris.Bar
Morris.Bar({
  element: 'graph',
  data: [
    {x: '2011 Q1', y: 3, z: 2, a: 3},
    {x: '2011 Q2', y: 2, z: null, a: 1},
    {x: '2011 Q3', y: 0, z: 2, a: 4},
    {x: '2011 Q4', y: 2, z: 4, a: 3}
  ],
  xkey: 'x',
  ykeys: ['y', 'z', 'a'],
  labels: ['Y', 'Z', 'A'],
  stacked: true
});
```
